# DysDiTect: Dyslexia Identification Using CNN-Positional-LSTM-Attention Modeling with Chinese Dictation Task

**DOI:** 10.3390/brainsci14050444

**Published:** 2024-04-29

**Authors:** Hey Wing Liu, Shuo Wang, Shelley Xiuli Tong

**Affiliations:** Human Communication, Learning, and Development (HCLD), Faculty of Education, The University of Hong Kong, Hong Kong 999077, China; hey0wing@connect.hku.hk (H.W.L.); shuowang@connect.hku.hk (S.W.)

**Keywords:** real-world applications, machine learning and dyslexia, handwriting, Chinese dictation task, sequence modeling

## Abstract

Handwriting difficulty is a defining feature of Chinese developmental dyslexia (DD) due to the complex structure and dense information contained within compound characters. Despite previous attempts to use deep neural network models to extract handwriting features, the temporal property of writing characters in sequential order during dictation tasks has been neglected. By combining transfer learning of convolutional neural network (CNN) and positional encoding with the temporal-sequential encoding of long short-term memory (LSTM) and attention mechanism, we trained and tested the model with handwriting images of 100,000 Chinese characters from 1064 children in Grades 2–6 (DD = 483; Typically Developing [TD] = 581). Using handwriting features only, the best model reached 83.2% accuracy, 79.2% sensitivity, 86.4% specificity, and 91.2% AUC. With grade information, the best model achieved 85.0% classification accuracy, 83.3% sensitivity, 86.4% specificity, and 89.7% AUC. These findings suggest the potential of utilizing machine learning technology to identify children at risk for dyslexia at an early age.

## 1. Introduction

Developmental Dyslexia (DD) is characterized by persistent difficulties in reading and phonological abilities [1], resulting in deficient decoding and spelling skills. In non-alphabetic languages such as Chinese, the writing system contributes to the multi-deficit nature of dyslexia [2]. Unlike most alphabetic words with linear letter sequences, Chinese characters have a multi-dimensional, multi-level feature set that includes orthography, phonology, and semantics at the character and radical levels, constructed using logographemes and strokes [3]. These multifaceted features intensify the complexity of potential writing errors, such as assimilation, substitution, insertion, deletion, and transposition at the radical and component levels, as well as protrusion, retraction, blending, segmenting, insertion, and deletion at the stroke level. These errors are frequently observed in copying and dictation tasks, which are the most common practices for children learning Chinese handwriting [4]. While the copying task measures motor ability, the dictation task directly assesses competence to accurately convert the pronunciation of spoken words into written form.

Behavioral studies suggest that Chinese DD children experience difficulties and delayed development in both tasks [5,6] due to impaired motor ability, phonological skills, and orthographic knowledge. The real-time analysis of handwriting performance demonstrated that these dyslexic individuals exhibited significantly more pause time and execution, as well as differences in pen pressure and character size [6], compared with their typically developing (TD) peers. Meanwhile, a similar analysis of Chinese dictation tasks showed the subtypes of handwriting difficulties and the association with lexical knowledge, perceptual-motor ability, and attention span in working memory systems [7]. Additionally, functional Magnetic Resonance Imaging (fMRI) studies clarify the neural basis for handwriting deficit in Chinese dyslexia by showing that DD children exhibited reduced activation in sensory-motor and visual-orthography processing but increased activation in executive control as a compensation mechanism [8]. Similarly, a follow-up study confirmed these patterns in brain network connectivity, with strength associated with handwriting speed [9].

Given the robust evidence of handwriting difficulties in Chinese DD children and the rapid advancements in machine learning technology, a crucial question arises: Can machine learning be used effectively to identify children at risk for dyslexia by analyzing their handwriting errors in a dictation task? This study presents a novel approach called **DysDiTect**, an automated **Dys**lexia **Di**ctation de**Tect**ion system that uses deep learning models and Chinese handwriting images in a dictation task to effectively classify individuals and predict their dyslexia status.

### 1.1. Technological Advancement of Chinese Handwriting and Performance Evaluation

The challenges faced by dyslexic individuals in handwriting have prompted the development of technological analysis and solutions. One such solution is handwritten Chinese character recognition (HCCR) technology, which has evolved from hierarchical and structural analyses to statistical modeling and deep-learning approaches [10]. For instance, optical character recognition (OCR) has surpassed human performances in recognizing handwritten characters. As HCCR and related innovations mature across languages, a focus on evaluating handwriting performance has increased in recent years.

One commonly used approach for assessing Chinese handwriting is statistical analysis of individual strokes [11,12]. This technique can provide detailed feedback on stroke quality, but it often fails to capture the overall representations of the character. To address this issue, previous studies have decomposed the structure of Chinese characters into quantifiable measurements and performed feature mapping with a standardized template for quality evaluation [13,14]. While feature mapping approaches have been effective, the complex algorithmic complexity used can lead to feedback that is incomprehensible to users, limiting their usage in educational settings.

The latest handwriting evaluation method [15,16] uses deep learning techniques to dissect and encode characters into smaller units of logographemes and assess the performances of each part [17]. This approach combines both structure-based and feature-mapping techniques, resulting in higher performance and informative feedback for users. Additionally, the system can be extended to implement stroke-based evaluations.

### 1.2. Transforming Dyslexia Identification: Transitioning from Human-Delivered Behavioral Tests to Machine Learning-Assisted Automatic Detection

The traditional approach for diagnosing DD involves a range of behavioral tests that assess various reading-related cognitive and meta-linguistic skills, such as the Hong Kong test of specific learning difficulties in reading and writing for primary school students (HKT-P) [18]. However, despite the compelling evidence highlighting the importance of handwriting analysis, this approach is considered inadequate due to its reliance on a limited number of tasks that may not fully capture the complexities of Chinese dyslexia. Furthermore, early identification and intervention of dyslexia are crucial for preventing adverse consequences [19]. However, the practical application of these measures is hindered by the high cost of, and labor-intensive effort required by, experienced educational psychologists and clinicians.

The rise of machine learning enables unconventional techniques and introduces new possibilities for early screening and identification of dyslexia. Extensive reviews [20,21,22] have examined the application of machine learning in dyslexia research, focusing on data sources, models/algorithms, feature selection, and evaluation metrics. Machine learning techniques for dyslexia identification utilize three main categories of data sources: behavioral symptoms, eye-tracking, and biomarkers [20]. Decades of research and clinical experiences have accumulated a large quantity of behavioral data related to the cognitive and language abilities of typically developing (TD) and DD children. Eye-tracking techniques have also enhanced our understanding of underlying cognitive processes of reading difficulties through the measurement of eye fixation. Additionally, different biomedical technologies such as fMRI, electroencephalography (EEG), and electrooculography (EOG) have been employed to investigate DD as a neurodevelopmental disorder. Depending on specific data sources, different machine-learning models are utilized [21]. Numerical (or preprocessed) data commonly use algorithms like Support Vector Machine (SVM), K Nearest Neighbors (KNN), Random Forest (RF), Decision Tree (DT), and regressions. Image data often incorporate deep learning techniques like Convolutional Neural Network (CNN).

Feature selection [22] is a critical step in machine learning aimed at identifying the most predictive features for improved prediction results and theoretical implications. Data preprocessing is sometimes employed to extract features from raw data, such as neuroimaging, brain signals, or handwriting metrics. To evaluate the performance and utility of machine learning models, various metrics are employed. Previous studies have reported accuracy rates ranging from 70–95% [20].

### 1.3. Predictions and Identification of Dyslexia Using Handwriting Features with Machine Learning Techniques

According to a recent review [22], approximately 30% of dyslexia prediction utilizing deep learning has been conducted using handwriting datasets. Previous attempts to identify dyslexia handwriting images have focused primarily on analyzing the basic unit of the writing system, namely, letters in alphabetic languages. DD is often manifested in prevalent errors such as reversed and corrected letters [23], as well as messiness in handwriting [24,25]. To facilitate the identification procedure, Optical Character Recognition (OCR) is incorporated, particularly in languages with a limited set of letters like English [26]. It is worth noting that dysgraphia identification research [27] has focused predominantly on studying the kinematic and static data of in-process handwriting, both in alphabetic languages and Chinese [28].

However, the techniques previously developed for dyslexia identification are not fully applicable to Chinese handwriting due to the multi-dimensional, multi-level features of Chinese characters. Lee et al. [29] utilized the error analysis of preprocessed dictation performance and successfully identified DD with an 80.0% accuracy rate, using stroke, grade, lexicality, and character configuration as the most predictive features. However, the labor-intensive nature of and reliance on knowledge-based expert coding of handwriting errors limited the practical implementation of this technique. As a result, a recent model called Dyslexia Prescreening Mobile Application for Chinese Children (DYPA) [17] utilizes deep learning encoding of multi-level features such as stroke, radical, and character to overcome these limitations and achieve an accuracy rate of 81.14% when combined with other meta-linguistic tests. It is important to highlight that DYPA was trained on a small dataset, including 39 Chinese DD children and 168 TD children in Grades 1–3. Such a small sample size may not fully reflect the variability and complexity of handwriting difficulties exhibited by Chinese DD children. More importantly, while DYPA achieved an accuracy rate of 81.14%, it is crucial to note that this was a result of combining handwriting analysis with other meta-linguistic tests. The extent to which handwriting analysis alone, without human expertise, can effectively differentiate DD and TD children remains unclear.

Thus, in this study, we advance previous research by developing DysDiTect, an automated Dyslexia Dictation deTection system that utilizes deep learning models and Chinese handwriting images in a dictation task to effectively classify individuals and predict their dyslexia status. To train and evaluate DysDiTect, we collected a large data set comprising 100,000 Chinese characters from 1064 children in Grades 2–6, including 483 DD and 581 TD children. Notably, our study is the first to employ deep learning techniques on handwriting images for identifying dyslexia in the Chinese language. We developed a series of models to evaluate the handwriting performances and temporal-sequential dependency of TD and DD children during Chinese dictation tasks.

## 2. Materials and Methods

### 2.1. Participants

Participants were 1064 native Cantonese-speaking school-age children taught to read and write traditional Chinese characters, with 483 formally diagnosed with dyslexia (Grade 2: *N* = 172; Grade 3: *N* = 110; Grade 4: *N* = 101; Grade 5: *N* = 72; and Grade 6: *N* = 28), and 581 typically-developing peers (Grade 2: *N* = 174; Grade 3: *N* = 158; Grade 4: *N* = 143; Grade 5: *N* = 62; and Grade 6: *N* = 44). The formal diagnosis of dyslexia was performed by educational or clinical psychologists using the Hong Kong test of specific learning difficulties in reading and writing for primary school students (HKT-P) [18].

### 2.2. Chinese Word Dictation Task

Adopted from HKT-P [18], this task required participants to write down in designated boxes 96 Chinese characters (48 two-character words) read aloud by the experimenter. Testing stopped after eight consecutive incorrect responses of two-character words, i.e., 16 characters.

### 2.3. Data Classification

The dataset consisted of scanned images of 869 handwritten encoded responses from Lee and Tong [3] and 195 handwritten raw responses. For the encoded data, each written Chinese character was binary-coded for multi-level, multi-dimensional features. Table 1 shows a summary of encoded data accuracies. A correct response meant that all written strokes, logographemes, and radicals within the character were accurately reproduced. A wrong response indicated to an incorrectly written structure within the character and blank, completely crossed out, or incomprehensible strokes not considered an attempt at writing. Cronbach’s *α* = 0.979.

### 2.4. Data Preprocessing

Each participant’s 96 handwritten Chinese character responses were color-scanned from the paper-based dictation test. Next, the images were cropped, isolated, and extracted from the designated boxes, then rescaled to a standardized size of 128 × 128 pixels of individual images, each containing a single Chinese character, resulting in 1064 × 96 = 102,144 images. The image size was selected to reduce computational cost while maintaining the details of strokes, which was confirmed by human inspection. A binarization operation was performed on each character image, converting the background to black and handwriting strokes to white to reduce computational cost, increase training speed, and decrease in-class variance. Notably, the experimental procedure and coding process would occasionally obstruct the handwritten responses with additional markings of ticks and crosses of some characters.

The preprocessing was completed using Python scripts with an automated edge detection technique. Additionally, manual checking and cropping were used to facilitate the dataset construction process. The training, validation, and test datasets were divided into an 8:1:1 ratio in a stratified grouping of both grade and dyslexic status, resulting in a sample size of 851:106:107 in the datasets.

### 2.5. Model Architecture

**Figure 1:**
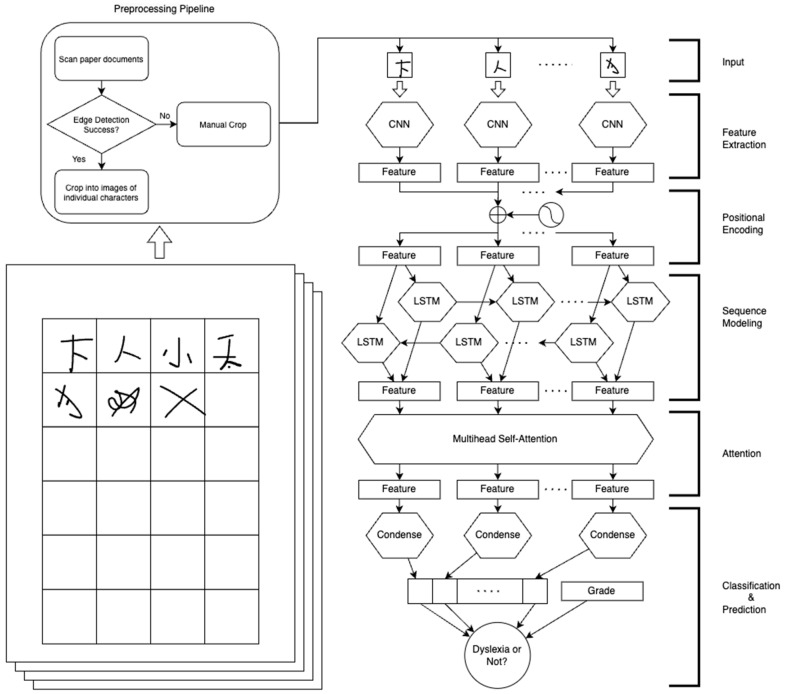
DysDiTect_PLAG model constructed for dyslexia prediction. Samples of participants’ handwritten responses are reproduced by the author and include correctly and incorrectly written characters, and visual-graphic symbols.

The model was adopted from existing deep learning architectures. First, the model utilized the independent characteristics of individual written characters by applying the feature extraction CNN module to every image and the positional encoding for incorporating the sequential properties in the dictation task. Then, the temporal-sequential dependency nature of the dictation task was captured using a stepwise LSTM module. Next, the self-attention layer was introduced to signify the feature maps. Finally, the Classification and Prediction module was used to predict the status of participants. The model architecture is shown in Figure 1.

#### 2.5.1. CNN Module with Positional Encoding

Convolutional neural network (CNN) is a type of deep learning model that is widely used in computer vision and image processing [30]. The CNN module used in this study consisted of convolutional layers and pooling layers [31]. The convolutional layers extracted features from images to adjust the training weight and bias of the neural network to generate the output feature maps of the input image [32]. The feature maps generated by convolutional layers could be connected to the next convolutional layer or pooling layers for feature extraction, or to a Fully Connected (FC) layer for classification. Moreover, pooling layers downsample the feature maps, reducing the computational costs while retaining the features learnt from the input image. 

The intrinsic features of Chinese handwritten characters were generalized into feature map representations. Each individual Chinese character of (3, 128, 128) was inputted into the CNN module and summarized as 32 neurons. Positional encoding [33] was introduced after the CNN module to leverage the positional information of the dictation sequence in the subsequent module. Then, the positional encoded feature map was passed to the LSTM module.

This module was adapted from the ResNet [34] architecture, which signifies the residual connection between convolutional layers to improve the performance of the model. The model built in this study adopted the ResNet-50 model, which consists of 50 layers, including convolutional and pooling layers.

#### 2.5.2. Bi-LSTM Module

A Long Short-Term Memory (LSTM) network is a type of Recurrent Neural Network (RNN) used for sequential data. This technique mimics the long-term and short-term memory systems in the human brain by implementing a gate system [35], that captures features and patterns within a time-series sequence [36]. Bi-directional LSTM (Bi-LSTM) considers the input sequence in both forward and backward directions, enabling longer dependency and the reversed order of features.

In the dyslexic prediction task based on the Chinese dictation task, the handwriting of characters followed a time sequence from the first character to the last character, which was suitable for LSTM. Thus, the temporal-sequential properties of handwriting characters were generalized and passed to the next attention module.

The feature maps from the CNN module extracted from each character were summarized as neurons and fed as input time steps into the LSTM. A 2-layer Bi-LSTM structure was used with 128 hidden states in each LSTM cell. The input data were encoded layer by layer. In each layer, the input data were encoded as a bi-directional connection of each cell both from the first to the last and from the last to the first in the Bi-LSTM structure. The final output of the LSTM cells was extracted and concatenated from both forward and backward directions as a feature map of the Fully Connected (FC) layer of 256 neurons.

#### 2.5.3. Multi-Head Self-Attention Module

In deep learning, the attention mechanism is considered one of the most important concepts and innovations [33], allowing each individual token to focus on different parts and “pay attention” to the input sequence. This mechanism signifies the importance of each token, enabling the model to selectively emphasize relevant information while downplaying irrelevant details, which overcomes the limitation of long-term dependency and enhances the model’s ability to capture complex relationships within the sequence [37].

With the intrinsic sequential properties of the dictation task being captured by the Bi-LSTM module, the integration of multi-head self-attention introduces a sophisticated mechanism for capturing cross-item linkages among handwritten characters. The attention context vector is then passed to the next classification and prediction module.

The feature map of each timestep from the Bi-LSTM module was passed to the 4-headed self-attention module. The dimensions of the final output of the attention module were unchanged, i.e., 256 neurons for each of 96 timesteps.

#### 2.5.4. Classification and Prediction with Grade Information

The output from the above modules served as the generalized representation of handwriting performance and behavior for the entire dictation task. Next, the embedding of each character was condensed into a single separate neuron and concatenated with the grade information to formulate the last FC layer consisting of 97 neurons. Finally, the FC layer was connected to a sigmoid activation function to predict whether the input was from TD or DD participants.

### 2.6. Model Training

Transfer learning in machine learning allows researchers to use state-of-the-art pre-trained models as the starting point, adapt to a specific problem or dataset, and enhance model performances and generalization capabilities [38]. The backbone CNN module was adapted from the ResNet-50 model, applied with pre-trained weights, and fine-tuned by the handwriting dataset for the dyslexia prediction task. Specifically, the pre-training on ImageNet was used for the well-established performances in previous studies by fine-tuning with a small dataset related to the downstream tasks [39]. In our model training, layer 4 and the FC layer were unfrozen for fine-tuning on the Chinese dictation dataset.

The models were built and trained with PyTorch and Lightning library on a Windows desktop with RTX 3060Ti 8 GB GPU. The minibatch sizes of 6 were used for model training, and the batches were reshuffled after each epoch. Adam optimizer with binary cross entropy was used to train the models. The learning rate was initially set as 5 × 10^−6^ and decreased by ×0.5 every three epochs. Regularization techniques were applied to avoid overfitting, where weight decay was set to 5 × 10^−5^, and dropout was set to 0.2 for LSTM, attention, and condensed layers. The models were trained for a maximum of 50 epochs with an early stopping setting when the validation loss did not improve for 1 × 10^−4^ in three consecutive epochs. A random seed of 42 was used for all settings.

## 3. Results

### 3.1. Pilot Study

Given that the dataset was derived from Lee and Tong [3] with encoded accuracies for each individual character, the authors first attempted to replicate previous approaches [40,41] for character-based predictions using the OCR/HCCR technique. However, the imbalanced classes of character accuracy (as reflected in Table 1) hindered the statistical power in evaluation metrics. The preliminary results were 95.7 ± 3.49% using a pre-trained model for the first 24 characters in 566 participants. However, data augmentation techniques would be required for further fine-tuning, but since they introduce doubt, decrease credibility of subsequent results, and, as shown by previous studies, do not capture the characteristics of dyslexic handwriting, we did not pursue their use.

### 3.2. Ablation Study

**Figure 2:**
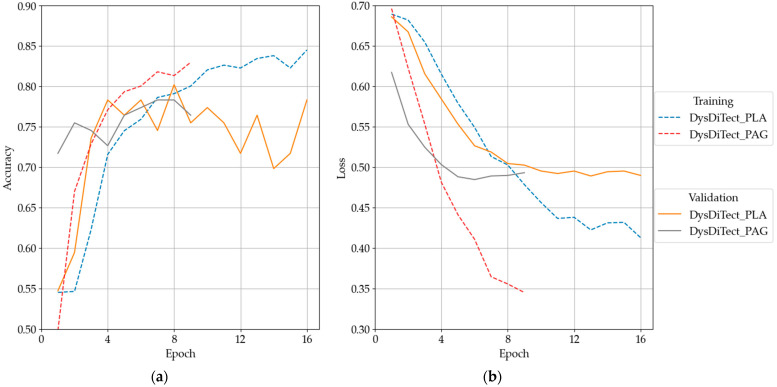
(**a**) Training and validation accuracy. (**b**) Training and validation loss.

The models were labeled as DysDiTect_{P/L/A/G}, where P refers to Positional encoding, L to LSTM, A to Attention, G to Grade, and brackets {} indicate optional modules. The modules/information were selectively dropped to verify the importance and usefulness of model design, resulting in a total of 16 models. Figure 2 shows the accuracies and losses of the training and validation set in DysDiTect_PLA and DysDiTect_PAG. Overfitting was observed when the training and validation loss diverged significantly. After training stopped, the test dataset was evaluated from the checkpoint with the lowest validation loss.

Table 2 shows the detailed results of the testing set, including the confusion matrix by lower (G2–3) and higher (G4–6) grades, with overall accuracy, sensitivity (correct rates of DD), specificity (correct rates of TD), and AUC. The best-performing model using only handwriting features is DysDiTect_PLA with 0.832 accuracy and 0.792 sensitivity. If grade information is included, the best-performing model is DysDiTect_PAG with 0.850 accuracy and 0.833 sensitivity. The confusion matrices revealed that higher grades have lower accuracy and sensitivity compared with lower grades in all models.

#### 3.2.1. Positional Encoding with Grade Information

With either (but not both) position encoding or grade information removed, the accuracy increased for the DysDiTect_PLAG model (0.776 to 0.832 [DysDiTect_PLA] and to 0.832 [DysDiTect_LAG]); the DysDiTect_PLG model (0.748 to 0.794 [DysDiTect_PL) and to 0.776 [DysDiTect_LG]); and the DysDiTect_PG model (0.710 to 0.738 [DysDiTect_P] and to 0.748 [DysDiTect_G]). With both position encoding and grade information removed, the accuracy increased for the DysDiTect_PLAG model from 0.776 to 0.822 (DysDiTect_LA); the DysDiTect_PLG model from 0.748 to 0.804 (DysDiTect_L); and the DysDiTect_PG model from 0.710 to 0.738 (DysDiTect_). These results suggested that the inclusion of this information may introduce unnecessary complexity and hinder the model’s ability to generalize effectively.

However, after removal of either or both position encoding and grade information, the opposite effect was observed for DysDiTect_PAG, where the accuracy decreased from 0.850 to 0.738 (DysDiTect_AG), 0.738 (DysDiTect_PA) and 0.766 (DysDiTect_A). These results suggested that the information is jointly learned by the Attention module.

#### 3.2.2. LSTM and Attention Modules

With the LSTM module removed, the accuracy decreased for most models except for DysDiTect_PLAG, which increased (0.776 to 0.850) compared with DysDiTect_PAG. The results conveyed the importance of the LSTM module in most situations, but also reflected its ability to obscure the Attention module when jointly learning both positional and grade information as mentioned above. Meanwhile, the removal of the Attention module resulted in decreased accuracies for most models except for DysDiTect_PA, which remained at 0.738 compared with DysDiTect_P, but decreased in AUC from 0.805 to 0.769. This result illustrated the importance of the Attention module, further analysis of which is discussed below.

### 3.3. Attention Map

**Figure 3:**
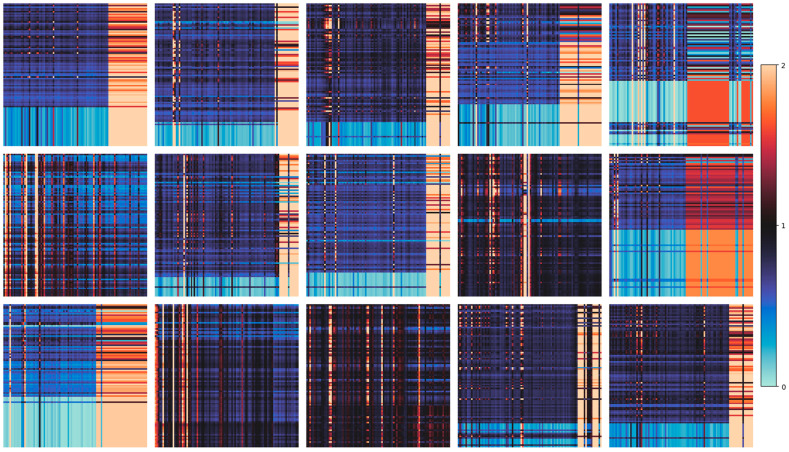
Examples of attention maps of DysDiTect_PAG.

**Figure 4:**
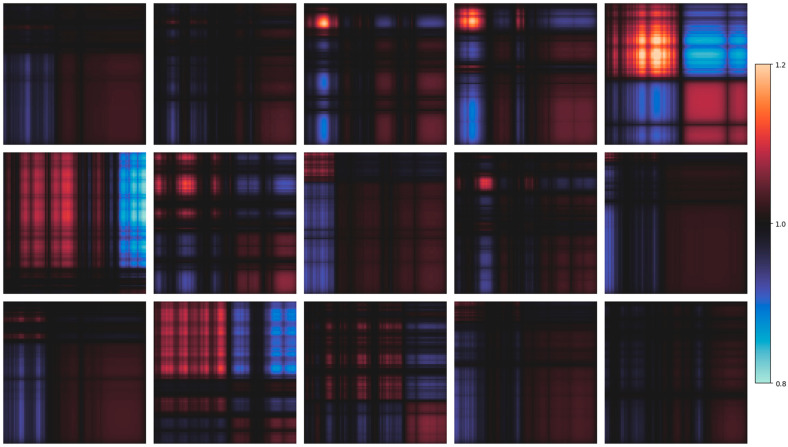
Examples of attention maps of DysDiTect_PLA.

The self-attention weights of DysDiTect_PAG and DysDiTect_PLA in the testing set were further evaluated. The examples are shown in Figure 3 and Figure 4, where the attention map from the same participant is listed in the same location. The order of sequences is ranked from high to low, with each row referring to the weight assigned to other tokens, and left to right, with each column referring to the weights assigned by other tokens. The weight scale is normalized by multiplying the sequence length of 96 characters and limiting it to (0, 2; Figure 3) and (0.8, 1.2; Figure 4) for visual representation.

The self-attention map of DysDiTect_PAG showed high variability of weights assigned to different tokens. Particularly, some tokens’ attained weights (i.e., the attention assigned by all tokens) were much higher than others, especially in later sequences for some participants (the continuous red columns on the right of attention maps). Notably, our observations diverged from those documented in prior studies, where the token-wise self-attention along the diagonal axis was not dominant.

The entropy of the attained weights in DysDiTect_PAG was calculated as the generalization of the randomness or uncertainty among individual characters. Table 3 shows the descriptive statistics by group and responses for the encoded participants in the testing dataset (*N* = 92; TD: *N* = 51; DD: *N* = 41).

The Intraclass Correlation Coefficients (ICC) between character entropy and accuracy measures were calculated. The ICC estimates and their 95% confidence intervals were calculated using Pingouin statistical package version 0.5.4 based on a mean-rating (k = 2), consistency, 2-way mixed-effects model. The overall ICC(3, k) = 0.418, 95%CI [0.12, 0.61], *F*(91, 91) = 1.72, *p* = 0.005. This result showed that the attained weights are correlated with the type of response, indicating that the weights of wrong responses were more uniformly assigned. Separating the correlations by groups, TD has ICC(3, k) = 0.512, 95%CI [0.14, 0.72], *F*(50, 50) = 2.05, *p* = 0.006; and DD has ICC(3, k) = 0.430, 95%CI [−0.07, 0.70], *F*(40, 40) = 1.75, *p* = 0.040.

During the character dictation task, the discontinuation criterion caused more wrong and blank responses in the later sequences. By focusing on those responses, the model could possibly identify the handwriting characteristics associated with dyslexia, e.g., reversed writings, radical substitution, stroke errors. This finding is consistent with previous studies [3,29] indicating that sublexical errors and responses are more predictive for identifying Chinese dyslexia.

In contrast, the self-attention weights of DysDiTect_PLA showed lower variations across the character sequence and were highly concentrated around the value of 1. Specifically, a prevalent characteristic across all attention maps was the absence of self-attention directed toward individual tokens themselves, though regional self-attention was observed. The majority of tokens exhibited relatively equal weights, suggesting a tendency towards uniform attention distributions across the input sequence. The attentions were mostly evenly distributed across multiple tokens or concentrated toward specific regions of the input sequence.

The attention map was based on the output of the LSTM module, where the intrinsic features of character sequences were already captured in the module. Therefore, self-attention was localized to amplify the generalized pattern of intrinsic characteristics of dyslexic handwriting.

## 4. Discussion

The experimental results demonstrated the robustness of DysDiTect with satisfactory performances. The proposed model framework is the proof of concept for a fully automated dyslexia screening system with a cost-effective solution. The Chinese dictation task lasted between 10 and 20 min, and the format was easily transformed to an electronic version to speed up the preprocessing pipeline. Furthermore, the technological advancement of faster algorithms [42] and hardware allowed real-time prediction to run directly on the user’s device. With the proposed system, teachers and parents can conduct self-screening to identify children at risk of dyslexia. Additionally, the in-process handwriting features can be incorporated for prediction performance.

Compared with previous studies using handwriting features for dyslexia identification via machine learning techniques, our results outperformed all evaluation metrics and were tested with an adequate sample size. The summary of results is shown in Table 4, which briefly lists the key information for evaluating performances. Notably, most results of previous studies were evaluated based on fragmented samples of data instead of the overall status of participants.

The ablation study evaluated the importance of the modules included in the constructed model. Surprisingly, the intertwined relationship between positional encoding and grade information obstructed the training process for most models. This result contradicts the Chinese dictation task’s incremental difficulty design [18], where some task items are expected to be acquired at higher grades. Meanwhile, based on ablation results, our detailed analyses demonstrated the usefulness of the LSTM and Attention modules and validated our model design.

Despite these satisfactory results, the black-box nature of the deep learning model limited our study’s explanatory power and theoretical evaluation. As such, the weights and biases in the model are generally not understandable or interpretable. Future research is encouraged to incorporate an explainable model for theoretical implications. Furthermore, although the handwriting deficit in Chinese dyslexia was assessed through the format of a dictation task, the underlying neurobiological mechanism has not been fully explored. Future studies are needed to investigate different task formats and language-specificity associated with aberrant neuroactivity in DD children, particularly in sensory-motor, visual-orthographical, and phonological processing. Finally, the precision of early diagnosis of dyslexia could be enhanced by synergizing machine learning techniques with neuroscience insights.

## 5. Conclusions

In this study, we proposed a novel approach to identify Chinese dyslexia: namely, using handwriting images with deep learning techniques in conjunction with a Chinese dictation task. The best-performing model, DysDiTect_PAG, achieved 85.0% classification accuracy, 83.3% sensitivity, 86.4% specificity, and 89.7% AUC. Using only handwriting features without grade information, the best-performing model, DysDiTect_PLA, achieved 83.2% classification accuracy, 79.2% sensitivity, 86.4% specificity, and 91.2% AUC. Future research may consider extending DysDiTect to different task formats and languages.

## Figures and Tables

**Table 1:** Performances on encoded Chinese dictation task (*N* = 869).

Grade		TD			DD		*t*	
	*N*	*M*	*SD*	*N*	*M*	*SD*		
2	165	42.9	15.4	162	20.9	12.4	14.20	***
3	111	53.1	16.7	101	32.6	16.2	9.06	***
4	102	68.1	13.6	54	38.3	16.5	11.40	***
5	56	76.3	13.0	62	48.9	18.9	9.26	***
6	31	83.0	10.3	25	56.3	17.4	6.76	***

Note. *** *p* < 0.001. Welch’s *t*-test was used. *N* = Number of participants. *M* = Mean accuracy. *SD* = Standard deviation of accuracy.

**Table 2:** Model prediction results of the testing set.

DysDiTect+				Confusion Matrix				Overall (*N* = 107)			
P	L	A	G	G23 (*N* = 62)		G456 (*N* = 45)		Accuracy	Sensitivity	Specificity	AUC
✓	✓	✓	✓	25	10	11	2	0.776	0.750	0.797	0.883
				3	24	9	23				
✓	✓	✓		24	6	14	2	0.832	0.792	0.864	0.912
				4	28	6	23				
✓	✓		✓	17	4	8	0	0.748	0.521	0.932	0.823
				11	30	12	25				
✓	✓			23	7	11	1	0.794	0.708	0.864	0.888
				5	27	9	24				
✓		✓	✓	26	4	14	4	0.850	0.833	0.864	0.898
				2	30	6	21				
✓		✓		22	8	7	1	0.738	0.604	0.847	0.805
				6	26	13	24				
✓			✓	15	3	5	0	0.710	0.417	0. 949	0.820
				13	31	15	25				
✓				21	5	10	6	0.738	0.646	0.814	0.769
				7	29	10	19				
	✓	✓	✓	24	6	14	2	0.832	0.792	0.864	0.922
				4	28	6	23				
	✓	✓		22	4	13	2	0.822	0.729	0.898	0.901
				6	30	7	23				
	✓		✓	25	9	9	1	0.776	0.708	0.831	0.833
				3	25	11	24				
	✓			22	4	9	0	0.804	0.646	0.932	0.855
				6	30	11	25				
		✓	✓	25	9	7	3	0.738	0.667	0.797	0.832
				3	25	13	22				
		✓		25	11	11	2	0.766	0.750	0.780	0.823
				3	23	9	23				
			✓	24	10	10	3	0.748	0.708	0.780	0.809
				4	24	10	22				
				21	9	10	2	0.738	0.646	0.814	0.858
				7	25	10	23				

Note. ✓ refers to the inclusion of modules; P = Positional encoding; L = LSTM module; A = Attention module; G = Grade information. Confusion matrices are listed as TP, FP, FN, TN (left to right, top to bottom) separately for Grades 2–3 and Grades 4–5–6. *N* = Number of participants. The best-performing metrics are highlighted in red and green for models with and without grade information, respectively.

**Table 3:** Descriptive statistics of entropy by group and response.

Group	Response		Entropy	
		*K*	*M*	*SD*
TD	Correct	3014	6.53	0.078
	Wrong	1882	6.49	0.101
DD	Correct	1235	6.51	0.098
	Wrong	2701	6.48	0.090

Note. *K* = Number of responses. *M* = Mean. *SD* = Standard deviation.

**Table 4:** Summary of results in previous studies.

Previous Studies	Language	Task		Sample Size		Dataset Size	Acc	Sen	Spe	AUC
				DD	TD					
Spoon et al. [24]	English	Patches from writing		11	77	25,650	0.557 ^a^	/	/	/
Spoon et al. [25]				22	78	/	0.776 ^a^	/	/	/
Isa et al. [26]	English/ Malaysian	4×	Letters + 4 × Digit	30	/	24	0.708 ^a^	/	/	/
Isa et al. [40]		Letters		/	/	39,897	0.870 ^a,b^	/	/	/
Rosli et al. [23]				/	/	233,354 × 0.2	0.953 ^a,b^	/	/	/
Yogarajah et al. [43]	Hindi	14×	Words from writing	54	/	267	0.861 ^a^	/	/	/
Jasira et al. [41]	English	Letters		/	/	86,115 × 0.1	0.950 ^a,b^	/	/	/
DYPA [17]	Chinese	Character copying + Behavioral tasks		39	168	/	0.811	0.743	0.827	0.790
Lee et al. [29]	Chinese	47×	Dictation	454	561	47,705	0.800	0.749	0.841	0.857
**DysDiTect_PLA**		96×		48	59	10,272	0.832	0.792	0.864	0.912
**DysDiTect_PAG**							0.850	0.833	0.864	0.900

Note. ^a^ Accuracies were based on fragmented samples (isolated letters, words, and patches). ^b^ Accuracies were based on OCR results of predefined classes (e.g., normal, reversal, corrected). “K ×” means the identical materials of size K were distributed to all participants. All sample sizes, dataset sizes, and metrics were based on the full dataset if cross-validation was used; otherwise, on the testing set only. Dataset size refers to the number or ratio of images used (if specified). Previous studies were ranked by author’s group and year of publication.

## Data Availability

The datasets presented in this article are not currently available due to privacy and confidentiality concerns for both participants and task materials.
